# Transition Diseases in Grazing Dairy Cows Are Related to Serum Cholesterol and Other Analytes

**DOI:** 10.1371/journal.pone.0122317

**Published:** 2015-03-25

**Authors:** Pilar Sepúlveda-Varas, Daniel M. Weary, Mirela Noro, Marina A. G. von Keyserlingk

**Affiliations:** 1 Animal Welfare Program, Faculty of Land and Food Systems, University of British Columbia, Vancouver, Canada; 2 Programa de Doctorado en Ciencias Veterinarias, Universidad Austral de Chile, Valdivia, Chile; 3 Hospital Veterinário, Universidade Federal do Pampa, Uruguaiana, Brazil; The University of Melbourne, AUSTRALIA

## Abstract

The objectives of this study were to describe the incidence of postpartum disease and to evaluate the association with serum cholesterol concentrations during the first 3 weeks after calving in grazing dairy cows. The association between non-esterified fatty acids (NEFA), β-hydroxybutyrate (BHBA), calcium and postpartum diseases was also evaluated. A total of 307 Holstein dairy cows from 6 commercial grazing herds in Osorno, Chile, were monitored from calving until 21 days in milk. Cases of retained placenta, clinical hypocalcemia and clinical mastitis were recorded by the farmer using established definitions. Twice weekly, cows were evaluated for metritis by the same veterinarian based on vaginal discharge and body temperature. Postpartum blood samples were collected weekly and analyzed for serum concentrations of cholesterol, NEFA, BHBA and calcium. Cows were considered as having subclinical ketosis if BHBA >1.2 mmol/L, and subclinical hypocalcemia if calcium <2.0 mmol/L in any of the 3 weekly samples. Overall, 56% of the cows studied developed at least one clinical or subclinical disease after calving. Incidence of individual diseases was 8.8% for retained placenta, 4.2% for clinical hypocalcemia, 11.7% for clinical mastitis, 41.1% for metritis, 19.9% for subclinical hypocalcemia and 16.6% for subclinical ketosis. Lower postpartum cholesterol in cows was associated with developing severe metritis or having more than one clinical disease after calving. For every 0.4 mmol/L decrease in serum cholesterol cows were nearly twice as likely to be diagnosed with multiple clinical diseases after calving. Higher BHBA concentrations and lower calcium concentrations during week 1 were associated with severe cases of metritis. Low serum calcium concentration during week 1 was also associated with developing more than one clinical disorder after calving. In conclusion, the incidence of postpartum diseases can be high even in grazing herds and lower serum cholesterol concentrations were associated with occurrence of clinical postpatum disorders.

## Introduction

During the transition from pregnancy to lactation increased energy and calcium demands for colostrum and milk production, combined with a decline in dry matter intake (DMI) around parturition, can result in negative energy balance (NEB), increased lipid mobilization [1,2] and a reduction in plasma concentrations of calcium [3,4]. These changes increase the risk of metabolic and infectious diseases after calving that are an animal welfare concern [5] and an important cause of production and economic losses to the dairy industry [6]. Although numerous studies have reported high rates of clinical and subclinical diseases after calving, most studies have focused on cows housed indoors and fed mixed rations [4,7,8]. Information on cows receiving the majority of their nutrients from pasture is more limited with high variability between studies [9–11], particularly concerning the frequency and method of disease recording.

Increased non-esterified fatty acids (NEFA) and β-hydroxybutyrate (BHBA) serum concentrations have been used as markers of excessive NEB and associated with increased risk of developing postpartum disorders [7,12–14]. Subclinical hypocalcemia around calving has been related to higher concentrations of NEFA, suggesting that normocalcemic cows are in better energy balance compared to subclinical hypocalcemic cows [4]. Although there is much evidence to support the use of NEFA and BHBA for predicting disease, few studies have explored whether other analytes related to energy metabolism may also be associated with postpartum health.

Serum cholesterol concentration in dairy cows generally decreases as parturition approaches but begins to increase gradually after calving [15], following the pattern of changes in DMI during this period [16]. Kim and Suh [17] reported that an energy deficit during the first month after calving as determined by severe body condition loss, was associated to lower cholesterol concentrations, suggesting that serum cholesterol may be a useful predictor of energy balance status during early lactation. Studies have examined the relationship between serum cholesterol concentration and disease but findings are inconsistent. For instance, Kaneene et al. [18] found an increased risk of retained placenta in cows with lower prepartum concentrations of cholesterol, while Quiroz-Rocha et al. [15] reported that the risk of retained placenta was higher with higher prepartum cholesterol concentrations. Thus, additional research is needed to determine if concentrations of cholesterol during the transition period may be useful to identify cows with an energy imbalance that predisposes them to postpartum diseases. The objectives of this study were to describe the incidence of postpartum diseases and to investigate their relationships with serum cholesterol concentrations during the first 3 weeks after calving in grazing dairy cows. The associations between NEFA, BHBA, calcium and postpartum diseases were also evaluated, as was the relationship between these metabolites and cholesterol.

## Materials and Methods

### Farm selection, cows and management

The University of British Columbia Animal Care Ethics Committee approved the methods described within this study [19]. This study was conducted on 6 seasonally calving commercial dairy farms in Osorno, Chile (40°34′ S, 73°9′ W). Selection of farms was not random and was a convenience sample of farms determined predominantly by time constraints as the maximum number that could be recruited and assessed within the spring calving period using a single trained veterinarian as the assessor. Those are the same farms used in Sepulveda-Varas et al. [20] and details on farm management are reported in that paper. The specific farms were selected with the help of a collaborating feed supplier using the following criteria: pasture based, milking > 200 cows, predominantly Holsteins and they were willing to participate. Average herd size was 302 ± 54 (± SD, ranging from 254 to 379) with milk production averaging 8,730 ± 1,090 kg/lactation (± SD, ranging from 7,329 to 9,650).

Data were collected during the spring calving season from July to December 2012. A convenience sample of 329 cows (62 primiparous and 267 multiparous) that calved during this time was selected with a median (range) parity of 3.0 (1 to 9). During the late-winter months of August and September, 25 and 142 cows were recruited, respectively. In the spring months of October and November, 120 and 42 cows were recruited. The mean ± SD number of cows enrolled per farm was 54 ± 10.7, with a range across all farms from 33 to 64 cows.

Transition period management consisted of a 60 d average dry period, and cows were moved from the far-dry group to the close-up group 24 ± 8.7 d (mean ± SD) before expected due date. Cows in the close-up group were kept on paddocks (5 farms) or in a barn (1 farm), all without access to fresh pasture. On all farms the close-up diet included a professionally formulated, commercially produced anionic transition supplement fed with hay and chopped straw. Immediately after calving, postpartum cows were moved to the lactating cow group and managed in a daily rotational grazing system typical for this region. Cows grazed a perennial ryegrass dominant pasture (*Lolium perenne*) that provided, on average, approximately 70% of the daily energy intake. Feeding was supplemented with grass silage according to the animals’ requirements. Concentrates (approximately 4 to 6 kg per cow per day) and a mineral mix (offered with the concentrate) were fed in the milking parlor during the morning and afternoon milking. All animals were milked twice daily between 0500 to 0800 and 1500 to 1800 h. With the exception of when they were milked, all cows were on pasture after calving.

### Data collection

Enrollment into the study occurred at calving. Cases of retained placenta and clinical hypocalcemia were recorded by the farm staff using established case definitions distributed and explained to the farm staff at the start of the experiment. Retained placenta was defined as failure to expel the placenta within 24 h after parturition. Clinical hypocalcemia was defined as any recumbent cow within 72 h after parturition exhibiting anorexia, nervous symptoms, staggering, varying degrees of unconsciousness, and good response to intravenously administered calcium. In addition, from calving to 21 days in milk (DIM), all cows were evaluated twice daily at the start of milking for signs of clinical mastitis by the milker. Clinical mastitis was characterized by the presence of abnormal milk or by signs of inflammation in 1 or more quarters.

Starting at d 3 until d 21 ± 1 after calving, all cows were monitored for metritis twice weekly, with 3 or 4 d between visits by a single trained veterinarian, using a manual vaginal examination and vaginal discharge (VD) score (following Huzzey et al. [21]). Appearance and smell of the VD was evaluated and assigned one of the following categories: no mucus or clear mucus = 0; cloudy mucus or mucus with flecks of pus = 1; mucopurulent (≤ 50% pus present) and foul smelling = 2; purulent (≥ 50% pus present) and foul smelling = 3; and putrid (red/brown color, watery, foul smelling) = 4. Cows were classified as having severe metritis if they had at least one VD score of 4 and one recording of fever (≥ 39.5°C). Cows were classified as having mild metritis if they had at least one VD score of 2 or 3 and no VD score of 4, with or without fever. During these health checks, presence of any other clinical disease(s), such as displaced abomasum, clinical ketosis, acute clinical mastitis or other clinical health disorder, were recorded.

Blood samples were collected postpartum between d 3 to 7 to represent wk 1, d 8 to 14 to represent wk 2, and d 15 to 21 ± 1 to represent wk 3. Blood was collected from the coccygeal vessel into 10-mL sterile tubes without an anticoagulant and then transported to the laboratory. Serum was separated immediately upon arrival at the laboratory, and the samples were stored at -20°C until analysis. Concentrations of BHBA (Ranbut, Randox, Crumlin, United Kingdom), NEFA (Randox, Crumlin, United Kingdom) and total cholesterol (CHOD-PAP, Human, Wiesbaden, Germany) were measured by enzymatic analysis using an auto-analyzer (Metrolab 2300, Wiener Lab, Rosario, Argentina). Intra- and inter-assay coefficient of variation (CV) for the BHBA assay were 2.8 and 4.7%, respectively, for the NEFA assay were 3.7 and 4.2%, respectively, and for cholesterol assay were 4.4% and 5.2%, respectively. Concentrations of calcium were determined using an atomic absorption spectrophotometer (Solaar Series S, Thermo Scientific, Electron Corporation, USA). Intra- and interassay CV for the calcium were 4.3% and 6.2%, respectively. Cows were considered as having subclinical ketosis if the BHBA concentration was of ≥ 1.2 mmol/L [8] in at least 1 of the 3 samples and clinical ketosis was not observed. Subclinical hypocalcemia was characterized as a concentration of Ca was <2.0 mmol/L [4,22] in at least 1 of the 3 blood samples and clinical hypocalcemia was not observed.

Diseases such as retained placenta, clinical hypocalcemia, clinical mastitis and metritis (mild and severe) were classified as clinical events. Subclinical ketosis and subclinical hypocalcemia were grouped as subclinical events. Cows were divided retrospectively into 3 health categories for statistical analyses: cows were categorized as having “no event” if they were never diagnosed with any type of disease; “one event”, included cows that were diagnosed with a single event of disease; or cow with “multiple events” when more than one disease was diagnosed. These categories were applied 3 times, considering only clinical, only subclinical or both clinical and subclinical. Incidence measures were calculated by health category and by individual event.

Of the 329 cows enrolled in the study, any cows showing evidence of systemic health problems (e.g., anorexia, depression), such as clinical ketosis (n = 3), acute clinical mastitis (n = 7) and digestive problems (n = 4) were not included in the analyses due the small number of cows that presented these disorders. Additionally, 3 cows that died and 5 that were culled during the study were excluded. These exclusion criteria yielded a total of 307 cows out of the pool of 329.

### Statistical analyses

Statistical analyses were performed using SAS software (version 9.3; SAS Inst. Inc., Cary, NC) with cow as the experimental unit. Descriptive statistics were generated with the FREQ statement.

To determine the association between serum analytes and clinical diseases, cows classified as having one clinical event were further categorized as having clinical mastitis only, mild metritis only and severe metritis only. Since no cows developed only retained placenta and just five cows developed only clinical hypocalcemia, these two diseases were not considered for the analytes analyzes as a single event category.

Analyte data were not complete for all cows. Values were available for 288 out of 307 cows in wk 1, 294 cows in wk 2 and for 289 cows in wk 3. Concentrations of certain blood analytes are known to change relative to time from calving, so data were stratified by period rather than analyzed using a repeated measures model [23]. Concentrations of NEFA, BHBA, cholesterol and calcium analytes were analyzed as continuous outcomes using PROC MIXED. The differences between no event cows versus cows with one clinical event and between no event cows versus cows with more than one clinical event were analyzed by period (wk 1, 2 and 3) using the estimate statement. The model considered farm as a random effect, parity (primiparous and multiparous), calving month (August, September, November and December) and health (no event, one event, multiple events) as fixed effects, and the interactions farm × health and parity × health. The covariance structures selected were compound symmetry and autoregressive based on the lowest Akaike’s information criterion. Residuals were examined to verify normality and homogeneity of variances and to detect possible outliers and influential points. No observations were removed from the analyses.

Data were further evaluated using logistic regression analysis to test if the various serum analytes (NEFA, BHBA, cholesterol and calcium) were associated to disease. The model included the fixed effect of parity and random effect of farm. Odds ratios (OR) were used to describe the level of association between the metabolite of interest and health outcome (no event cows versus one event and no event cows versus more than one event). Analytes were tested separately for each week, versus health outcomes over the entire postpartum period.

The CORR procedure of SAS was used to determine Pearson correlation coefficients between NEFA, BHBA, cholesterol and calcium analytes at each week after calving.

Differences with p ≤ 0.05 were considered significant and 0.05 < p ≤ 0.10 were considered as a tendency.

## Results

### Incidence of postpartum diseases

The number and proportion of cows that were allocated to the 3 defined health categories are shown in Table 1, stratified by farm and parity. Considering only subclinical diseases, 27.4% of the cows presented one event (hypocalcemia or ketosis) and 4.5% presented more than one (both hypocalcemia and ketosis). Considering only clinical diseases (retained placenta, clinical hypocalcemia, clinical mastitis, severe or mild metritis), 30.3% and 15.3% of the cows presented only one event or more than one event, respectively. Considering both clinical and subclinical health events, 24.8% of the cows presented only one event and 31.3% two or more events. Only 44.0% of the cows were not diagnosed with any clinical or subclinical disease event postpartum.

**Table 1:** Incidence of clinical and subclinical events during the first 21 DIM by farm (A, n = 63; B, n = 53; C, n = 59; D, n = 53; E, n = 49; F, n = 30) and parity [primiparous (PP), n = 57; multiparous (MP), n = 250], including overall incidence (n = 307).

Disease and health status	Overall		Farm						Parity	
	No.	%	A	B	C	D	E	F	PP	MP
Clinical diseases										
Type of disease										
Retained placenta	27	8.8	12.7	13.2	5.1	0	14.3	6.7	2.0	6.8
Clinical hypocalcemia	13	4.2	7.9	7.6	1.7	0	2.0	6.7	0.3	3.9
Clinical mastitis	36	11.7	19.1	13.2	10.2	0	14.3	13.3	3.3	8.5
Mild metritis	73	23.8	22.2	39.6	27.1	9.4	22.5	20,0	7.5	16.3
Severe metritis	53	17.3	15.9	17.0	15.3	10.2	20.4	26.1	3.9	13.4
Health status										
No event	167	54.4	54	39.6	54.2	77.4	49	50	35.1	58.8
One event	93	30.3	27	39.6	33.9	22.6	30.6	26.7	42.1	27.6
Multiple events	47	15.3	19	20.8	11.9	0	20.4	23.3	22.8	13.6
Subclinical diseases										
Type of disease										
Hypocalcemia	61	19.9	31.8	24.5	20.3	9.4	12.2	16.7	4.2	15.6
Ketosis	51	16.6	7.9	26.4	25.4	11.3	14.3	13.3	3.6	13.0
Health status										
No event	209	68.1	63.5	58.5	59.3	84.9	73.5	73.5	63.2	69.2
One event	84	27.4	33.5	32.1	35.6	9.9	26.5	23.5	31.6	26.4
Multiple events	14	4.5	3.1	9.4	5.1	5.6	0	3.3	5.3	4.4
Clinical and subclinical										
Health status										
No event	135	44.0	34.9	30.2	40.7	69.8	44.9	46.7	21.0	49.2
One event	76	24.8	33.3	30.2	22	18.8	18.4	23.3	40.4	21.2
Multiple events	96	31.3	31.8	39.6	37.3	11.4	36.7	30.0	38.6	29.6

### Serum analytes and postpartum diseases

Table 2 shows analyte concentrations in serum for wk 1, 2 and 3 after calving by disease status. NEFA and BHBA concentration were greater in cows with severe metritis relative to cows that had no clinical event during wk 1 only (p = 0.04 and p = 0.003, respectively). Cholesterol concentrations were lower in cows diagnosed with mild and severe metritis during wk 2 (p = 0.01 and p < 0.001, respectively) and wk 3 (p = 0.04 and p = 0.03, respectively), and lower in all periods after calving for cows that developed multiple events relative to cows in the no clinical event category (wk 1, p = 0.04; wk 2, p < 0.001; wk 3, p < 0.001). The concentrations of calcium were lower in cows that developed multiple events than the no clinical event cows during wk 1 only (p < 0.001). Additionally, concentrations of calcium tended to be lower during wk 1 in cows with severe metritis (p = 0.08) and during wk 2 in cows with clinical mastitis than those in cows with no clinical event (p = 0.08).

**Table 2:** Least squares mean (±SE) serum NEFA, BHBA, cholesterol and calcium concentrations in grazing cows relative to health outcome during the first 3 wk after calving.

Analyte and health outcome		Period relative to calving		
	n	wk 1	wk 2	wk 3
NEFA (mmol/L)				
No event	153	0.60 ± 0.05	0.59 ± 0.06	0.46 ± 0.10
Clinical mastitis	14	0.68 ± 0.10	0.69 ± 0.10	0.49 ± 0.12
Mild metritis	54	0.58 ± 0.06	0.57 ± 0.08	0.42 ± 0.10
Severe metritis	22	0.76 ± 0.09*	0.70 ± 0.10	0.51 ± 0.10
Multiple events	45	0.70 ± 0.06†	0.58 ± 0.08	0.49 ± 0.12
BHBA (mmol/L)				
No event	153	0.65 ± 0.04	0.72 ± 0.04	0.66 ± 0.04
Clinical mastitis	14	0.64 ± 0.09	0.73 ± 0.09	0.68 ± 0.04
Mild metritis	54	0.73 ± 0.05	0.73 ± 0.06	0.65 ± 0.05
Severe metritis	20	0.96 ± 0.08***	0.88 ± 0.08	0.60 ± 0.05
Multiple events	45	0.74 ± 0.05	0.74 ± 0.06	0.64 ± 0.05
Cholesterol (mmol/L)				
No event	153	2.15 ± 0.17	2.70 ± 0.20	3.18 ± 0.20
Clinical mastitis	14	2.20 ± 0.20	2.39 ± 0.20†	3.12 ± 0.20
Mild metritis	54	2.21 ± 0.18	2.42 ± 0.20**	2.96 ± 0.20*
Severe metritis	20	2.29 ± 0.20	2.18 ± 0.20**	2.83 ± 0.20*
Multiple events	45	1.94 ± 0.18*	2.25 ± 0.20***	2.81 ± 0.20**
Calcium (mmol/L)				
No event	153	2.29 ± 0.04	2.34 ± 0.04	2.38 ± 0.04
Clinical mastitis	14	2.28 ± 0.08	2.22 ± 0.06†	2.29 ± 0.06
Mild metritis	54	2.26 ± 0.06	2.34 ± 0.04	2.38 ± 0.04
Severe metritis	20	2.17 ± 0.08†	2.26 ± 0.06	2.38 ± 0.06
Multiple events	45	2.08 ± 0.06***	2.30 ± 0.05	2.36 ± 0.04

^a^ No event = cows that did not develop any clinical disorder of interest (retained placenta, clinical hypocalcemia, clinical mastitis, mild or severe metritis) by 22 DIM.

^b^ Multiple events = cows that developed 2 or more clinical disorders (retained placenta, clinical hypocalcemia, clinical mastitis, mild or severe metritis) by 22 DIM.

†*P* ≤ 0.1

*P ≤ 0.05

**P ≤ 0.01

***P ≤ 0.001 indicate differences in these analytes between No event cows and cows with one event (clinical mastitis, mild metritis or severe metritis only), and between No event cows and cows with Multiple events.

Table 3 shows that high BHBA concentrations in wk 1 (p = 0.001) and wk 2 (p = 0.063) were associated with developing severe metritis. Low cholesterol concentrations were associated with a developing severe metritis during wk 2 (p = 0.002) and wk 3 (p = 0.100), and low cholesterol in each of the three wks (and no other analyte) was also associated with having multiple health events (wk 1, p = 0.003; wk 2, p = 0.002; wk 3, p = 0.005). Low calcium was also associated with developing multiple events, but only for measures taken during wk 1 (p <0.001). Postpatum NEFA was never associated with having clinical events after calving.

**Table 3:** Logistic regression models describing the association between serum NEFA, BHBA, cholesterol and calcium in grazing cows and the risk for the health outcomes of interest: (1) severe metritis versus no event, and (2) multiple events versus no event during the first 3 wk after calving.

Period relative to calving and health outcome	Analyte	OR^a^	95% CI	*P-*value
wk 1				
Severe metritis	NEFA	1.00	1.00–1.01	0.122
	BHBA	1.95	1.32–2.90	0.001
	Cholesterol	0.85	0.67–1.08	0.199
	Calcium	1.40	0.99–1.99	0.060
Multiple events	NEFA	1.00	1.00–1.01	0.002
	BHBA	1.12	0.78–1.61	0.514
	Cholesterol	1.54	1.16–2.04	0.006
	Calcium	1.83	1.39–2.40	<0.001
wk 2				
Severe metritis	NEFA	1.00	1.00–1.00	0.374
	BHBA	1.34	0.98–1.83	0.063
	Cholesterol	1.69	1.20–2.36	0.002
	Calcium	1.23	0.85–1.79	0.266
Multiple events	NEFA	1.00	1.00–1.00	0.220
	BHBA	1.03	0.79–1.33	0.805
	Cholesterol	1.81	1.40–2.38	<0.001
	Calcium	1.20	0.90–1.58	0.196
wk 3				
Severe metritis	NEFA	1.00	1.00–1.00	0.901
	BHBA	0.85	0.53–1.37	0.535
	Cholesterol	1.22	0.95–1.54	0.100
	Calcium	0.91	0.62–1.32	0.632
Multiple events	NEFA	1.00	1.00–1.00	0.226
	BHBA	1.57	0.98–2.52	0.606
	Cholesterol	1.44	1.17–1.77	0.008
	Calcium	1.18	0.86–1.60	0.287

^a^Adjusted odds ratio per 0.1 unit increase in NEFA (mmol/L), per 0.3 unit increase in BHBA (mmol/L), 0.4 unit decrease in cholesterol (mmol/L), and per 0.2 unit decrease in calcium (mmol/L).

Results are shown separately for the 3 wk after calving.

During wk 1 and wk 2 after calving, NEFA and BHBA were positively correlated (r = 0.24 and 0.19; p < 0.001). NEFA was negatively correlated with cholesterol during each period after calving (r = −0.22, −0.15, and −0.16 for wk 1, 2 and 3; p < 0.001). Moreover, NEFA was negatively correlated with calcium during all periods (r = −0.26, −0.19 and −0.22; p < 0.001). Cholesterol was positively correlated with calcium during all periods (r = 0.23, 0.33, and 0.21 for wk 1, 2, and 3, respectively; p ≤ 0.001). Neither cholesterol nor calcium was correlated with BHBA in any wk after calving (p > 0.18).

## Discussion

### Incidence of postpartum diseases

Our results are consistent with other work showing that clinical and subclinical disease after calving are common in grazing dairy cows [9,11,24]. Incidence appears to be similar to that observed for cows housed indoors [7,23]. The incidence of retained placenta, mastitis, metritis, and subclinical ketosis and hypocalcemia varied across herds as others have reported for confinement dairy farms in Finland and North America [25] and in Australian grazing dairy herds [24]. This variation in incidence of post-partum diseases is likely a consequence of different herd management practices and skill at controlling each of these conditions [24]. Incidence may also depend on the quality of herd health records. In our study, cases of retained placenta, clinical hypocalcemia and mastitis were obtained from farm records, which could have influenced our findings.

Incidences of retained placenta (8.8%) and clinical mastitis (11.7%) in our study were similar to that previously reported for grazing dairies [11,24]. An earlier Chilean study reported incidences of 15.6% for retained placenta and 14.4% for mastitis in confinement systems [26]. Clinical hypocalcemia (4.2%) was more common than the 2% incidence reported for seasonally calving New Zealand farms [9]. Earlier work on confinement herds has also reported variable rates. For example, Melendez et al. [26] found a clinical hypocalcemia incidence of 5.2% and Chapinal et al. [7] an incidence of 2.4%. Although we used standardized case definitions and explained their use to all farm staff, it is likely that some of the farm-to-farm variation in the current study was due to differences in how individual farm staff applied these definitions.

The incidence of severe metritis (17.3%) in our study was similar to that reported previously for large confinement herds (17.6% [27]; 16.1% [28]) but higher than that previously reported for grazing systems (5.7% [11]). The diagnosis of metritis is challenging and inconsistent on commercial dairies [29] and is likely influenced by the transition management protocols. For instance, in the study of Ribeiro et al. [11] cows were evaluated for metritis on d 7 and 14 postpartum. Thus brief cases, which did not coincide with a day of testing, may have been missed resulting in an underestimation of the true incidence. In our study, bi-weekly monitoring by the same veterinarian likely allowed for better detection of the condition. To our knowledge, no other studies have reported the incidence of metritis in early lactation grazing dairy cows that were followed using an intensive protocol similar to that used in the present study.

The high incidence (24%) of mild metritis is a cause of concern. Metritis negatively affects milk production, reproduction and culling. Mildly and severely metritic cows produced on average 6 and 8 kg less milk per day during the first 3 wk of lactation compared to healthy cows [21]. Wittrock et al. [30] found that metritic cows failed to recover in milk production for the first 20 wk of lactation, despite being treated, and found that these sick cows were also more likely to be culled from the herd. Several studies have reported a reduction in pregnancy rates and increase in days open in cows diagnosed with uterine infection during the first 3 wk after calving [31–33].

Incidences of subclinical ketosis range from 9 to 43% in the first 2 months of lactation [8,34,35] with the highest risk occurring within the first 21 d of lactation [36,37]. McArt et al. [8] found that the peak risk of subclinical ketosis occurred at 5 DIM. Prevalence of subclinical ketosis in the first 2 wk after calving (i.e., blood BHBA ≥1.2 mmol/L) in 528 dairy herds from 10 European countries, averaged 22% and ranged from 11 to 37% [38]. In two grazing farms, Ribeiro et al. [11] report an overall prevalence of 35%, based on BHBA concentration ≥0.96 mmol/L. However, the previous studies (as in the current study) tested cows only weekly. Taking into consideration that the median time to resolution of subclinical ketosis is 5 days [8] testing less frequently than twice per week will underestimate the occurrence of this disease.

The incidence of postpartum subclinical hypocalcemia in the current study varied from 9 to 32% (average 20%) in the six farms visited. This incidence is lower than that reported for previously for grazing herds (43%; [11]) and confinement herds (25 to 54%, depending on parity [4]). Differences among studies may relate to differences in blood sampling. For instance, Ribeiro et al. [11] only analyzed calcium during the first wk postpartum (d 7 ± 3) versus weekly for 3 wk in the current study. Normal reference values for Ca concentrations in the serum of cattle have been defined as 2.0 to 2.5 mmol/L [22,39], but different cutoff values for subclinical hypocalcemia have been used (≤1.8 mmol/L [40]; ≤2.0 mmol/L [39] and ≤2.14 mmol/L [41]). Reinhardt et al. [4] definition of hypocalcemia was similar to the <2.0 mmol/L used in this study, but measures were limited to the first 48 hours after calving. The lowest serum Ca concentration typically occurs within the few days after calving [40,41], and returns to normal within 2 to 3 d [42,43]. We were unable to measure calcium before 3 DIM, likely resulting in an underestimate of this ailment.

### Serum analytes and postpartum diseases

The results of the current study showed that low concentrations of cholesterol are associated with postpartum health disorders, especially severe metritis and developing more than one clinical event after calving. Kaneene et al. [18] and Kim and Suh [17] also found that cows with lower serum cholesterol concentrations were more likely to developed metritis after calving.

The mechanism of association between lower cholesterol concentration and illness after calving is not entirely clear but work by others [16,17,44] suggests that this association is related to blood cholesterol levels being related to (1) DMI and (2) to energy balance status after calving, as both decreased DMI and NEB are linked to illness in dairy cows [21,45]. Although the cause-and-effect relationship between DMI, metabolic and health status is difficult to determine from data collected postpartum [18], the metabolic change observed in cows during the study is likely a consequence of the illness, as cows showed signs of disease concurrent with the decrease in cholesterol concentration.

As previously mentioned, serum cholesterol concentration has been associated with DMI in healthy transition dairy cows [16], likely because in ruminants most of circulating triglyceride-rich lipoproteins are of intestinal origin [46,47]. Several studies have shown that sick cows consume less feed compared to healthy cows after calving [21,48,49]. Indeed, Huzzey et al. [21] showed that cows with severe and mild metritis ate approximately 5 kg/d and 3 kg/d less DM, respectively, compared with healthy animals throughout the 3-wk postpartum. In a recent study, cows with clinical mastitis after calving reduced DMI by 1 kg/d DM in the few days before diagnosis, but rebounded on the day following intramamary treatment [50]. In the present study, failure to show an association between cholesterol and mastitis may be explained by mastitic cows maintaining their DMI.

NEB plays a critical role in many diseases during transition [51], and severe NEB has been linked to decreased blood cholesterol concentrations after calving [17,52]. Prepartum NEFA concentrations are a more accurate measure of NEB than ketone bodies and are more predictive of negative health outcomes than BHBA (reviewed by McArt et al. [53]). In our study, cholesterol was negatively correlated with serum NEFA but not with BHBA during the first 3 wk after calving, suggesting that lipid mobilization, NEFA and cholesterol concentrations in serum are interrelated. Elevated NEFA concentrations can be associated with reduced liver function because of re-esterification of fatty acids and accumulation of triglycerides in the cytoplasm of hepatocytes, potentially leading to a further decrease in DMI [51]. Other work has found lower concentrations of cholesterol in cows with fatty infiltration of the liver (reviewed by Van Saun [54]). However, these relationships are complex perhaps accounting for the low correlation between NEFA and cholesterol in the current study.

The association between NEB (as indicated by high NEFA and BHBA around calving) and postpartum disease has been documented in a few earlier studies [45,55] but not in others [26,41]. Hammon et al. [45] and Galvão et al. [55] found that high BHBA and NEFA persisted until the second and fourth wk of lactation in cows with metritis, respectively, whereas Martinez et al. [41] reported that cows that developed metritis did not have higher concentrations of NEFA and BHBA after calving. In our study, we found that high concentrations of BHBA in the first 2 wk after calving were associated with severe metritis, but found no association between postpartum NEFA or BHBA concentration and the occurrence of multiple disease events. However, our findings do suggest a relationship between health status and concentrations of cholesterol. On the basis of these results and in agreement with Kim and Suh [17], we suggest that low serum cholesterol is a better indicator of NEB during early lactation and a better predictor of postpartum diseases.

A limitation of this study was our inability to determine cholesterol concentrations during the weeks before calving. This limitation reduces our ability to understand the association between prepartum cholesterol and disease outcome. Previous work has shown that lower DMI prepartum is a risk factor for cows that develop post-partum diseases (i.e. metritis) relative to cows that remain healthy [21,45,56]. We suggest that future studies include measures of cholesterol during the prepartum period to assess the usefulness of this analyte in predicting post-partum disease.

## Conclusions

The incidence of clinical and subclinical diseases after calving can be high on pasture-based farms. Lower serum cholesterol was associated with the occurrence of severe metritis and multiple disease events, suggesting that this is a useful metabolite for monitoring health status after calving.
